# Dynamic Adsorption Properties of Insoluble Humic Acid/Tourmaline Composite Particles for Iron and Manganese in Mine Wastewater

**DOI:** 10.3390/ma15124338

**Published:** 2022-06-19

**Authors:** Ling Liu, Jiadi Ma, Xiaowan Yu, Tianyi Zhang, Vitumbiko Mkandawire, Xilin Li

**Affiliations:** 1 School of Civil Engineering, Liaoning Technical University, Fuxin 123000, China; majiadi0924@163.com (J.M.); yuxiaowan5158@163.com (X.Y.); zty18342846242@163.com (T.Z.); vitumbikomkandawire@rocketmail.com (V.M.); lixilin@lntu.edu.cn (X.L.); 2 Information Industry Electronics Eleventh Design and Research Institute Technology Engineering Co., Ltd., Dalian Branch, Dalian 116000, China; 3 Water Services Association of Malawi, Tikwere House, City Center, Private Bag 390, Lilongwe 207213, Malawi

**Keywords:** dynamic adsorption, insoluble humic acid, iron, manganese, mine wastewater, tourmaline, Thomas model

## Abstract

Iron- and manganese-contaminated mine water is widespread around the world, and economical and efficient remediation has become a priority. Insoluble humic acid/tourmaline composite particles (IHA/TM) were prepared by combining inorganic tourmaline (TM) with the natural organic polymer humic acid (HA), and the effects of different calcination temperatures and calcination times of TM and IHA on the adsorption of Fe^2+^ and Mn^2+^ were analyzed. Based on the microscopic characterization of Scanning electron microscopy (SEM), Energy Dispersive Spectroscopy (EDS), Brunnauer–Emmet–Teller (BET), X-ray diffractometer (XRD) and Fourier transform infrared (FTIR), the simultaneous adsorption performance of IHA/TM on Fe^2+^ and Mn^2+^ was studied through dynamic adsorption tests, and a dynamic adsorption model was established. Adsorption regeneration experiments were carried out to further investigate the effectiveness of the composite particles in practical applications. The results show that, when the calcination temperature was 330 °C and the calcination time was 90 min, the removal rates of iron and manganese by the IHA/TM composite particles reached 99.85% and 99.51%, respectively. The curves for penetration of Fe^2+^ and Mn^2+^ ions into the IHA/TM composite particles were affected by the bed height, flow rate and influent concentration. Decreasing the flow rate, decreasing the influent concentration, or increasing the bed height prolonged the operation time of the dynamic column. If the bed height was too low, the penetration point was reached before the expected treatment was achieved, and when the bed height was too high, the removal of Fe^2+^ and Mn^2+^ was slow, and the utilization rate of the adsorbent was also reduced. If the flow rate was too low, longitudinal remixing easily occurred in the column. However, when the flow rate was too high, the speed of Fe^2+^ and Mn^2+^ ions passing through the adsorption layer increased, which reduced the total amount of adsorption. The increase in influent concentration not only reduces the removal rate, but also greatly shortens the total operation time of the dynamic column and reduces the treatment water. The dynamic process for the adsorption of Fe^2+^ and Mn^2+^ by IHA/TM was fitted best by the Thomas model. The adsorption column was continuously regenerated five times, and the results show that the IHA/TM composite particles were suitable for iron and manganese removal from mine wastewater. The research results will provide a reference for the effectiveness of the IHA/TM composite particles in practical applications.

## 1. Introduction

Mining leads to the dissolution of minerals containing iron and manganese in rocks, the discharge of iron- and manganese-contaminates industrial wastewater and the infiltration of leaching water from the ore-dressing waste slag accumulation site into the underground well, which leads to high concentrations of iron and manganese in the groundwater of the mining area [1]. This mine wastewater with high concentrations of iron and manganese is directly discharged without treatment, which poses a serious threat to human health and the environment, especially in the vast water-deficient mining areas where groundwater is the source of drinking water [2]. After the occurrence of Fe^2+^ and Mn^2+^ ions, groundwater appears reddish-brown in color, with special odor and turbidity problems; it can also cause chronic poisoning when ingested in excess over time [3,4]. In India, Nepal, the Philippines, Denmark, as well as many mining areas in eastern Inner Mongolia, Yunnan and Guizhou, Shanxi Datong, Henan Hebi, among others in China, the problem of iron and manganese exceeding the standard levels exists, causing great distress to residents who use groundwater as their drinking water source [5]. The average concentrations of Fe^2+^ and Mn^2+^ in abandoned neutral and weakly alkaline mine wastewater in South Korea were 25.5 mg/L and 2.06 mg/L, respectively [6]. The Fe^2+^ and Mn^2+^ concentrations of mine water samples from a deactivated coalmine in the southern State of Santa Catarina, Brazil, were 112.79 mg/L and 3.64 mg/L, respectively [7]. The mass concentrations of Fe^2+^ and Mn^2+^ in the mine water of the Badaohao Coal Mine in Jinzhou, Liaoning, China, were 35.5 mg/L and 13.2 mg/L, respectively [8]. The iron content of the mine water of Dafeng Mine in Shandong, China can reach up to 239.2 mg/L, and the manganese content also reaches 11.3 mg/L [9]. The limits for Fe^2+^ and Mn^2+^ concentrations in drinking water set by the World Health Organization are 0.3 mg/L and 0.1 mg/L, respectively, beyond which the water needs to be treated.

The removal method of iron and manganese in mine wastewater is basically the same as that in groundwater, such as oxygenation and aeration, contact oxidation, manganese sand-filter material filtration, ion exchange, microfiltration and adsorption [10,11]. Compared with other pollutant treatment methods for groundwater, the adsorption method has the advantages of good treatment effect, simple process, low price, strong practicability and environmental friendliness and has become a frequent choice for iron and manganese removal [12,13]. Nassar et al. used corncob as adsorbent to treat 1 mg/L iron and manganese ions, and the removal rates were 79% and 81%, respectively [14]; Bright et al. used synthesized zeolite Y to remove iron and manganese ions from groundwater, and found that zeolite could remove 98% of 0.2 mg/L iron ion and 97% of 0.05 mg/L manganese ion within 1 h [15]. In addition, for low concentrations of metal ions, such as iron and manganese ions, in groundwater, various adsorbents, such as diatomite, serpentine, hydroxyapatite, and biochar, have attracted the attention of researchers and achieved good adsorption effects [16,17]. However, the removal effect of high-concentration iron- and manganese-contaminated groundwater is indeed unsatisfactory.

As an inorganic compound, tourmaline adsorbs heavy metal ions to the negative electrode of the crystal through the electrostatic field on the surface of the crystal and combines with OH^−^ to form basic salt or precipitation, which does not cause secondary pollution, and tourmaline can be used repeatedly, which shows it is an excellent adsorption material [18]. However, due to the presence of a large amount of OH^−^ on the surface of tourmaline, tourmaline has a high hydrophilic surface and surface energy, which makes it very easy to agglomerate, difficult to separate from water and easy to block the adsorption pores. The removal of heavy metals alone often fails to meet the requirements of water quality monitoring, and surface modification of organic matter or preparation of organic–inorganic composite materials can reduce tourmaline agglomeration and improve the adsorption efficiency of heavy metals. Liao et al. successfully deposited silver nanoparticles on the surface of polyacrylic-acid-modified tourmaline by in situ reduction. The introduction of polyacrylic acid can greatly improve the dispersibility of tourmaline in water, and PAA-modified Tm/Ag composites have high adsorption potential for dyes and heavy metals [19]. Wei et al. prepared tourmaline–bamboo charcoal ceramic composites with inorganic tourmaline and organic bamboo fibers, which can effectively remove hexavalent chromium from wastewater [20]. Humic acid (HA) is a high-molecular-weight organic substance containing various functional groups. It has high reactivity and a loose “sponge-like structure”, which can adsorb, complex and redox with heavy metals in the environment to form stable chelates [21]; it is also an organic material with strong heavy metal adsorption potential. The composite of humic acid and inorganic materials can make full use of the respective advantages of the two materials to enhance the adsorption of heavy metal ions. Leone V et al. [22] adsorbed humic acid onto zeolite tuff and prepared a zeolite–humic acid adduct by heat treatment. The results showed that the heat treatment induced the decarboxylation of HA, which reduced its solubility to water and ensured effective immobilization on tuff and effective adsorption of organic matter. Hu et al. [23] loaded humic acid onto hydroxyapatite to make HA/HAP composite adsorbents, which efficiently adsorbed Cd^2+^ from water. Zhang et al. [24] combined humic acid with Fe_3_O_4_ to synthesize a novel adsorbent for the adsorption of methylene blue. The results showed that HA–Fe_3_O_4_ nanoparticles adsorbed methylene blue efficiently and could be reused.

Judging from the current research status, there are few studies on the removal of high-concentration iron- and manganese-contaminated mine wastewater by adsorption, and the adsorption capacity of the existing adsorbents is limited, which cannot achieve the desired effect. The inorganic material tourmaline with self-generating polarity is compounded with the organic material humic acid containing a variety of active groups, and the advantages of organic–inorganic composite materials are fully utilized to prepare a new insoluble humic acid/tourmaline composite particles (IHA/TM) with good adsorption effect. It will be a very meaningful attempt to use the new IHA/TM composite material for the adsorption of high-concentration iron- and manganese-contaminated mine wastewater.

Based on this background, a new low-cost IHA/TM composite adsorbent is prepared in this study. The prepared composite particles have a porous spatial structure and are insoluble in water, which not only solves the dissolution problem of HA, but also changes the surface properties and environmental behavior of tourmaline, improves the stability and reactivity of tourmaline and reduces its surface agglomeration, which enhances the removal of pollutants. Through indoor dynamic experiments, the simultaneous adsorption of Fe^2+^ and Mn^2+^ by the composite particles was studied to provide a reference for the application of IHA/TM composite adsorbents in mine wastewater pollution remediation in mining areas.

## 2. Materials and Methods

### 2.1. Experimental Water Quality

The mass concentrations of Fe^2+^ and Mn^2+^ in the simulated mine wastewater samples were 25 mg/L and 10 mg/L, respectively, and the pH was 6. The Fe^2+^ and Mn^2+^ concentrations were measured by a Z-2000 atomic absorption spectrophotometer, and the pH was measured by a PHS-3C precision pH meter.

### 2.2. Preparation and Characterization of Materials

#### 2.2.1. Preparation of the IHA/TM Composite Particles

Tourmaline was obtained from black tourmaline (iron tourmaline) in Liaoning, China, and crushed to a particle size smaller than 48 μm (passing No. 300 ASTM sieve) with a crusher for use. The chemicals used in the experiments, such as humic acid, etherified starch, sodium hydroxide and hydrochloric acid, were purchased from Beijing Kangpu Huiwei Technology Co., Ltd.(Beijing, China). The reagents were all of AR analytical grade.

**Figure 1:**
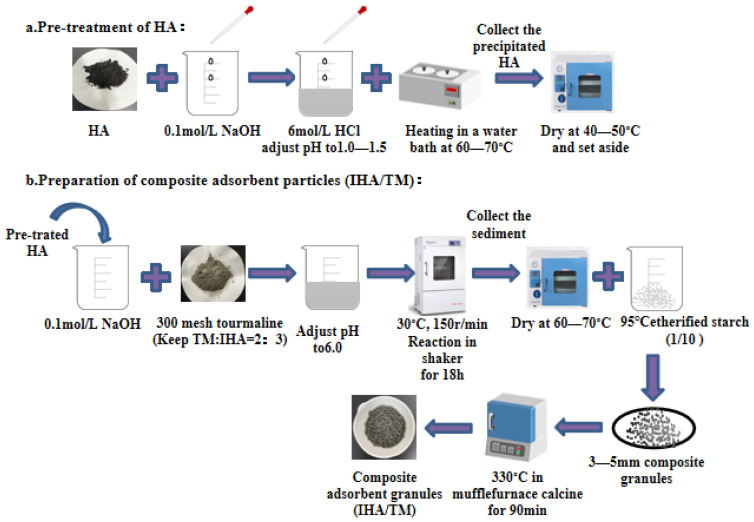
Preparation process of IHA/TM.

Preparation of IHA/TM composite particles: IHA/TM composite particles were prepared according to the reference of our research group [25]. First, insoluble humic acid (IHA) was prepared, and then the optimal preparation conditions for IHA/TM composite particles were determined by investigating different mixing ratios of TM:IHA (1:3, 2:3, 1:1, 3:2 and 3:1), mixing times (6 h, 12 h, 18 h, 24 h and 30 h), calcination temperatures (240 °C, 270 °C, 300 °C, 330 °C and 360 °C) and calcination times (30 min, 60 min, 90 min, 120 min, 150 min and 180 min) on the removal effect of Fe^2+^ and Mn^2+^. The preparation process is shown in Figure 1.

#### 2.2.2. Micro Test Analysis

The specific surface area and pore size of the IHA/TM composite particles were analyzed by Quanta Atuosorb-iQ specific surface area and porosity analyzer (Boynton Beach, FL, USA). The surface morphology and chemical element types of the composite particles were tested by JSM-7500F scanning electron microscope (Akishima, Japan) and FYFS-2002E EDS detector (Waltham, MA, USA). The phase analysis was performed using Shimadzu XRD-6100 X-ray diffractometer (Kyoto, Japan) and MDI jade 6.0 software to determine and analyze phase data and the chemical structure analysis of the composite particles was performed using IRPrestige-21 Fourier transform infrared spectrometer (Kyoto, Japan).

### 2.3. Dynamic Adsorption Experimental Equipment

**Figure 2:**
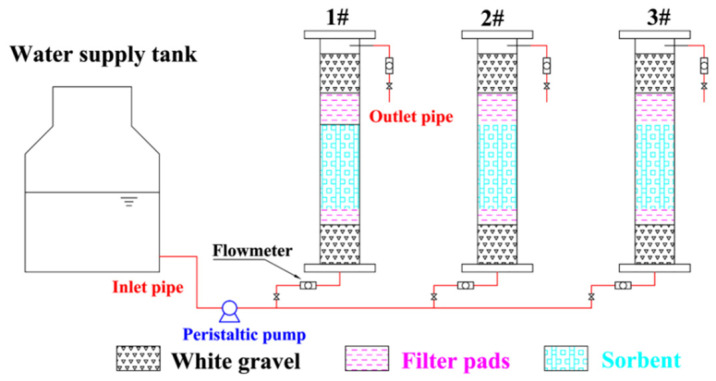
Schematic diagram of the dynamic adsorption device. (1#, 2#, and 3# in the figure are the dynamic adsorption columns under different reaction conditions.)

The temperature was 23 ± 2 °C, and the relative humidity was 50 ± 5% in the laboratory. The test was carried out in an organic glass column with an inner diameter of 41 mm and a height of 25 cm. In addition, 4 mm of white gravel and 2 mm of filter cotton were added as protective layers at both ends to achieve an even flow and prevent the loss of adsorbent. The water inlet and outlet mode of the device was down–in and up–out; the water sample was lifted using a BT50S peristaltic pump (Norcross, GA, USA), and the flow was controlled by an LZB-6 glass rotameter. To prevent Fe^2+^ from being oxidized, deionized water was used to discharge air. Before the experiment, the deionized water was slowly injected from the bottom to the top with a peristaltic pump to exclude air in the column, and during the experiment, water samples were filled with composite adsorbent voids, and a liquid level height of 2 cm was maintained above the gravel layer. The samples were sampled at the sampling port according to set sampling times. The concentrations of Fe^2+^ and Mn^2+^ in raw water and effluents were determined. The influence of different column heights, flow rates and influent concentrations on the adsorption process was investigated. The column height and influent flow rate were determined based on the optimal adsorbent dosage, reaction time, column length and column diameter determined from previous single-factor experiments, and the gradient was obtained by moderate extension on both sides of the middle value in the actual project. The influent concentration takes into account the actual situation in Fuxin, Liaoning, China, and the maximum value was determined by referring to the maximum concentration of water quality in high iron and manganese areas in China. The adsorption test device is shown in Figure 2. All water quality determination experiments were performed in three groups of parallel experiments. The error value is the standard deviation.

### 2.4. Experimental Data Analysis

The adsorption capacity and removal rate *R* (%) of the adsorbent per unit mass of Fe^2+^ and Mn^2+^ were calculated as follows:

(1) $$R=\frac{C_{0}-C_{e}}{C_{0}}\times 100\%$$

where *C*_0_ is the initial solution concentration, mg/L; and *C*_e_ is the solution concentration at adsorption equilibrium, mg/L.

The penetration curve was plotted as the ratio of pollutant effluent concentration *C*_t_ to influent concentration *C*_0_ with operating time *t*. The penetration point was set at the time when the effluent concentration was 5% of the influent concentration (*C*_t_/*C*_0_ = 0.05), and the depletion point was set at the time when the effluent concentration was 95% of the influent concentration (*C*_t_/*C*_0_ = 0.95); the corresponding times were the penetration time (*t*_b_) and depletion time (*t*_e_). The total amount of ions adsorbed by the dynamic column (*q*_total_) was calculated according to Equation (2), and the adsorption capacity *q*_e_ was calculated according to Equation (3) [26].

(2) $$q_{\mathrm{total}}=\frac{Q}{1000}\int_{0}^{t}(C_{0}-C_{t})d_{t}$$

(3) $$q_{e}=\frac{q_{\mathrm{total}}}{m}$$

where *Q* is the inlet water volume flow rate, mL/min; and *m* is the composite adsorbent mass, g.

### 2.5. Penetration Curve Model Fitting

The Adams–Bohart model, Thomas model and Yoon–Nelson model were selected to simulate dynamic processes for the adsorption of Fe^2+^ and Mn^2+^ by IHA/TM composite particles.

The Adams–Bohart model is more used to describe the adsorption characteristics in the initial region of the penetration curve, allowing us to better understand the characteristic parameters, such as the mass transfer rate constant *K*_AB_ and the saturation concentration N_0_ of ion adsorption [27]. The expression is shown in Equation (4).

(4) $$\ln\left(\frac{C_{t}}{C_{0}}\right)=K_{\mathrm{AB}}C_{0}t-K_{\mathrm{AB}}N_{0}(\frac{H}{W})$$

where *K*_AB_ is the Adams–Bohart mass transfer rate constant, L/(mg-min); *H* is the loading column height, cm; *W* is the solution flow rate, cm/min; *N*_0_ is the ion adsorption saturation concentration, mg/L; and *t* is the working time, min.

The Thomas model is widely used to analyze the change characteristics of adsorption performance when there are different parameters, such as bed depth, flow rate and concentration [28]. It is expressed by Equation (5).

(5) $$\ln\left(\frac{C_{0}}{C_{t}}-1\right)=\frac{K_{\mathrm{Th}}q_{0}m}{Q}-K_{\mathrm{Th}}C_{0}t$$

where *K*_Th_ is the Thomas mass transfer rate constant, mL/(min·mg); *q*_0_ is the maximum adsorption capacity of the adsorbent, mg/g; and *m* is the mass of the composite adsorbent filled in the dynamic column, g.

The Yoon–Nelson model is a semiempirical model that is fitted without considering the adsorption flow rate and adsorbent dosage; this minimizes the errors arising from the use of the Thomas model, especially at lower or higher time periods of the penetration curve [29]. The expression is shown in Equation (6), and the theoretical adsorption amount is calculated in Equation (7).

(6) $$\ln(\frac{C_{t}}{C_{0}-C_{t}})=K_{\mathrm{YN}}(t-\tau )$$

(7) $$q_{\mathrm{YN}}=\frac{C_{0}vt}{1000m}$$

where *K*_YN_ is the adsorbate rate constant, min^−1^; *τ* is the time for 50% of adsorbate to penetrate the dynamic column, min; and *q*_YN_ is the theoretical unit adsorption amount, mg/g.

### 2.6. Regeneration of IHA/TM

After the dynamic adsorption experiment was completed, the IHA/TM composite particle column bed was regenerated with 1 mol/L HNO_3_ at a flow rate of 10 mL/min, and then the bed column was washed with deionized water until the pH was close to neutral. The EDS spectrum scanning of TM/IHA composite particles was carried out between the regeneration cycles, and the relative content of iron and manganese on the material was determined. The adsorption–desorption process was repeated 5 times to evaluate the reusability of the IHA/TM composite particles.

## 3. Results and Discussion

### 3.1. Preparation of IHA/TM Composite Particles

**Figure 3:**
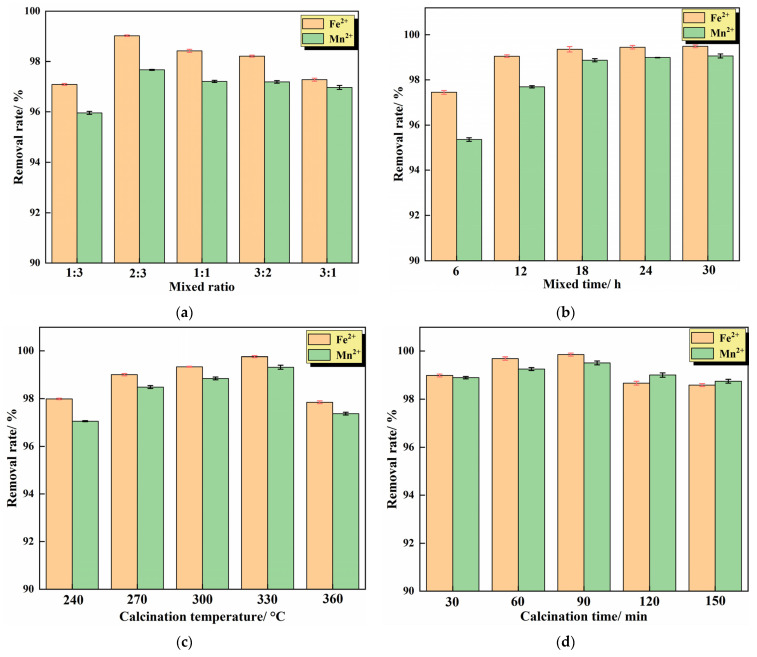
Preparation conditions of the IHA/TM composite particles: (**a**) mixing ratio; (**b**) mixing time; (**c**) calcination temperature; and (**d**) calcination time. (Other conditions are subject to optimal conditions).

The IHA/TM was prepared according to Section 2.2.1. The effect of different reaction conditions on the removal of Fe^2+^ and Mn^2+^ was analyzed, and the experimental results are shown in Figure 3.

#### 3.1.1. Effect of Mixing Ratio

It can be seen from Figure 3a that the IHA/TM composite particles exhibit different adsorption behaviors for Fe^2+^ and Mn^2+^ at different ratios. With the increase in the proportion of TM, the removal rates of Fe^2+^ and Mn^2+^ by composite particles showed a trend of increasing first and then decreasing. This is because when the proportion of TM in the composite increases, the ability to release negative ions increases, the adsorption performance is enhanced and the removal rate of Fe^2+^ and Mn^2+^ on the composite increases with the increase in TM proportion. However, when the proportion of TM is further increased, the TM in the composite cannot be sufficiently dispersed, the TM may be in agglomerated state and the adsorption properties cannot be effectively exerted. A certain amount of IHA can improve the dispersion performance of TM and reduce its agglomeration. At the same time, a large number of active groups (such as -COO-, -COOH and -OH) on the surface of IHA can transfer electrons, which can improve the adsorption capacity. However, when the proportion of IHA is too high, it also occupies the active sites of TM, resulting in a decrease in the removal rate of iron and manganese. Therefore, there is an optimal TM:IHA ratio, and it was determined experimentally that the adsorption performance is the best when the mixing ratio of TM and IHA is 2:3. Chen et al. also found a similar phenomenon when using tourmaline and montmorillonite to prepare composite materials to adsorb Pb(II); if the ratio of tourmaline and montmorillonite is too high or too low, it is not conducive to the synthesis of composite materials, and when the proportion of tourmaline is 30.7%, the synthetic composite material has the best removal effect of Pb(II) [30].

#### 3.1.2. Effect of Mixing Time

The ratio of TM to IHA was set to 2:3, the mixing time was controlled according to the preparation method, and the effect of mixing time on the removal effect of the composite was investigated. The experimental results are shown in Figure 3b. It can be seen from Figure 3b that, with the prolongation of mixing time, the removal rate of Fe^2+^ and Mn^2+^ by the IHA/TM composite particles increases gradually. When the mixing time of TM and IHA was 18 h, the removal rates of Fe^2+^ and Mn^2+^ were 99.36 ± 0.23% and 98.87 ± 0.31%, respectively. After the mixing time exceeds 18 h, the removal rate increases slowly and tends to be stable. With the increase in mixing time, tourmaline was uniformly dispersed into the IHA solution with a larger specific surface area, which reduced the agglomeration of TM. During the experiment, it was found that the composite adsorbent prepared with the mixing times of 24 h and 30 h absorbed a large amount of water due to the long mixing time, and the gasification phenomenon occurred during the subsequent calcination, resulting in the breakage of the adsorbent particles and the increase in effluent turbidity. Considering the effluent effect and economy comprehensively, it was appropriate to determine the mixing time of 18 h.

#### 3.1.3. Effect of Calcination Temperature

The ratio of TM to IHA was set to 2:3, the mixing reaction was performed for 18 h, the calcination temperature was controlled according to the preparation method, and the effect of the calcination temperature on the removal of iron and manganese from the composite was investigated. The experimental results are shown in Figure 3c. Figure 3c shows that the removal rate of iron and manganese by IHA/TM composite particles is the largest at 330 °C, and the removal rate decreases when the temperature is higher or lower than 330 °C. This is because thermally activated tourmaline has a large specific surface area and negatively charged surface, which can improve the adsorption performance of metal ions [18]. However, if the processing temperature is higher than the thermal stability of tourmaline, the lattice may be partially destroyed, the internal structure may collapse, and the surface iron can be oxidized from divalent to trivalent, thereby increasing the positive charge on the surface [31]. The adsorption capacity of Fe^2+^ and Mn^2+^ would be weakened by the enhancement of electrostatic repulsion and the natural polarity of tourmaline [32]. In addition, studies have shown that 330 °C is the optimum temperature for humic acid heat treatment. The humic acid particles after the 330 °C treatment decrease, the pores increase, and the removal efficiency is the highest at this time. When the temperature exceeds 330 °C, humic acid is sintered and carbonized, reducing the internal acid group and reducing the adsorption performance of humic acid itself [33]. Therefore, 330 °C was determined to be the optimal calcination temperature.

#### 3.1.4. Effect of Calcination Time

The ratio of TM to IHA was set to 2:3, the mixing reaction was performed for 18 h, and the calcination temperature was controlled at 330 °C to prepare composite particles according to the preparation method. The effect of calcination time on the removal effect of the IHA/TM composite was investigated. The experimental results are shown in Figure 3d. Figure 3d shows that the removal effects of the IHA/TM composite particles synthesized with different calcination times on Fe^2+^ and Mn^2+^ in the water samples first increased and then decreased. The removal rate of Fe^2+^ and Mn^2+^ by the synthesized IHA/TM composite particles reached the maximum when the calcination time was 90 min. The attractiveness of IHA increased over time, while the TM decreased over time after starting heating at 330 °C. The combination of the two reached a maximum at 90 min. After 90 min, it reaches a level where the drop in TM exceeds the enhancement achieved by IHA. Zhao et al. [34] used IHA to remove Mn^2+^ in an aqueous solution, indicating that too long a calcination time would result in the partial loss of -COOH and -OH functional groups in IHA, reducing the number of available metal binding sites on IHA. Therefore, the time for the high-temperature modification of composite particles should not be too long. Therefore, the time for the high-temperature modification of the composite particles should not be too long. The optimal calcination time was determined to be 90 min, and the corresponding removal rates of Fe^2+^ and Mn^2+^ were 99.85 ± 0.12% and 99.51 ± 0.31%, respectively.

#### 3.1.5. Comparison of Adsorption Effects of Different Adsorbents on Fe^2+^ and Mn^2+^

**Figure 4:**
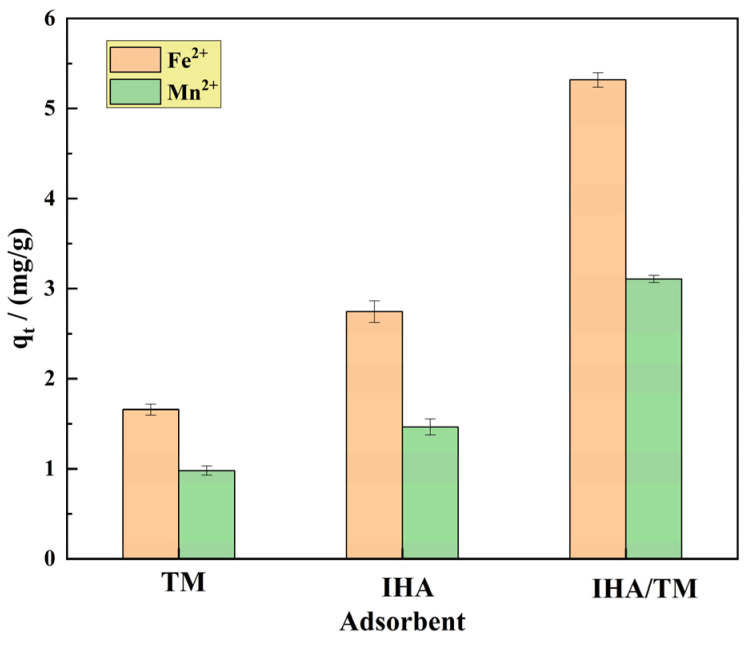
The effects of different materials on the adsorption of Fe^2+^ and Mn^2+^.

To confirm the excellent properties of the composites, comparative experiments were carried out using TM, IHA and IHA/TM. The TM and IHA samples were also calcined at 330 °C for 90 min in the control experiment in order to ensure the comparable adsorption of the materials. The TM, IHA and IHA/TM composite materials (1.0 g) were taken to remove 100 mL of Fe^2+^ and Mn^2+^ solutions with contents of 10–100 mg/L, respectively. The pH was controlled at 6.0, the temperature was controlled at 25 °C, and the samples were passed through a 0.45 μm filter membrane after adsorption equilibrium. The Fe^2+^ and Mn^2+^ concentrations were measured in three groups of parallel experiments, and the results are shown in Figure 4. Figure 4 shows that the equilibrium adsorption capacities of TM, IHA and IHA/TM for the adsorption of Fe^2+^ are 1.657 mg/g, 2.740 mg/g and 5.318 mg/g, respectively. For the adsorption of Mn^2+^, the equilibrium adsorption capacities of TM, IHA and IHA/TM are 0.982 mg/g, 1.461 mg/g and 3.106 mg/g, respectively, indicating that the removal capacity of IHA/TM for Fe^2+^ and Mn^2+^ is significantly better than that of TM and IHA. The removal rate of Fe^2+^ by IHA/TM was 3.21 times and 1.94 times higher than that of TM and IHA, respectively. The removal rate of Mn^2+^ by TM/IHA was 3.16 times and 2.13 times higher than that by TM and IHA, respectively. This shows that after the two materials are combined, the two materials have a promoting effect, and IHA increases the dispersion properties and active sites of TM, thereby significantly enhancing the reactivity.

Table 1 is a comparison of the adsorption capacity of iron and manganese ions of some adsorbent materials mentioned in the literature. Table 1 shows that the IHA/TM composite particles have a much higher simultaneous adsorption capacity for iron and manganese than similar adsorption materials and have strong application potential as an adsorbent for iron and manganese removal.

#### 3.1.6. Effect of Coexisting Ions

**Figure 5:**
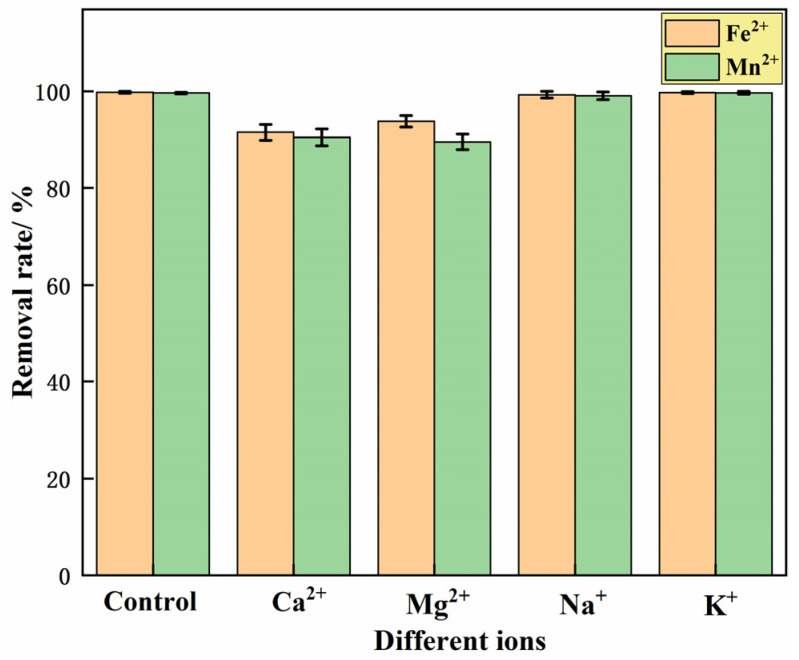
The effect of co-existing ions on the Fe^2+^ and Mn^2+^ removal effect.

To investigate the effect of co-existing cations (Ca^2+^, Mg^2+^, Na^+^ and K^+^) in iron- and manganese-contaminated groundwater in mining areas on the adsorption of iron and manganese ions by the IHA/TM composite particles, the concentrations of Ca^2+^, Mg^2+^, Na^+^ and K^+^ were set to be the same as the initial iron ion concentration, which was 25 mg/L. The co-existing ions were added into 100 mL water samples containing Fe^2+^ or Mn^2+^, and then 1 g IHA/TM composite particles were added. The removal rates of Fe^2+^ and Mn^2+^ were measured in a shaking table (25 °C, 150 r/min) after 240 min. The results are shown in Figure 5. Figure 5 shows that the removal efficiency of Fe^2+^ by IHA/TM is reduced by 8.49 ± 1.62%, 6.23 ± 1.27%, 0.71 ± 0.72% and 0.25 ± 0.21%, respectively, after adding Ca^2+^, Mg^2+^, Na^+^ and K^+^ in the water samples containing Fe^2+^. The removal efficiency of Mn^2+^ by IHA/TM is reduced by 9.51 ± 1.70%, 10.44 ± 1.65%, 0.93 ± 0.85% and 0.31 ± 0.27%, respectively, after adding Ca^2+^, Mg^2+^, Na^+^ and K^+^ in the water samples containing Mn^2+^. This shows that Na^+^ and K^+^ in water have almost no effect on the adsorption of Fe^2+^ and Mn^2+^ ions by IHA/TM, while Ca^2+^ and Mg^2+^ have a certain inhibitory effect on the adsorption of Fe^2+^ and Mn^2+^ by IHA/TM. Overall, the IHA/TM composite particles have strong anti-interference ability for the removal of Fe^2+^ and Mn^2+^.

### 3.2. Composite Material Characterization

**Figure 6:**
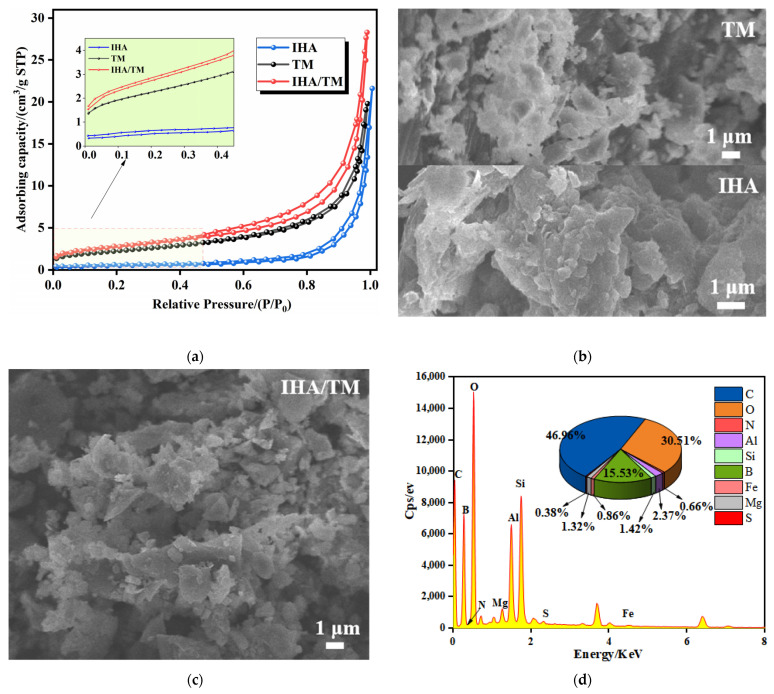

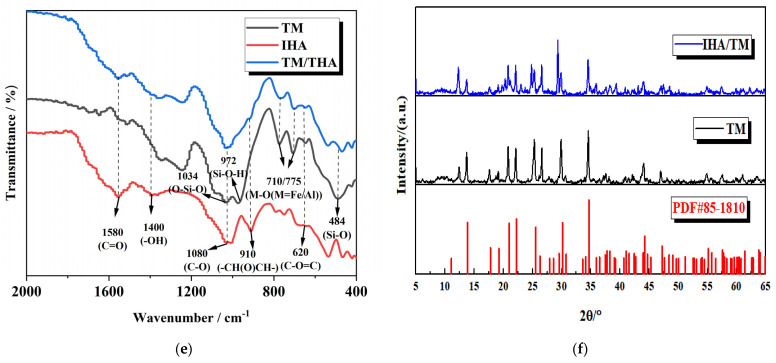
Micro analysis. (**a**) N_2_ adsorption–desorption isotherms of TM and IHA/TM; (**b**) SEM images of TM and IHA; (**c**) SEM images of IHA/TM; (**d**) EDS spectra of IHA/TM; (**e**) FTIR spectra of IHA/TM; and (**f**) XRD patterns of IHA/TM.

N_2_ adsorption–desorption isotherms for TM and IHA/TM are shown in Figure 6a. From the comparison of adsorption capacity, the higher the position of the adsorption curve, the more pores there are in the material, indicating that the composite material improves the porosity of the material. The specific surface area, average pore volume and average pore size of TM are 8.08 ± 0.12 m^2^·g^−1^, 0.0031 ± 0.0003 cm^3^·g^−1^ and 2.53 ± 0.06 nm, respectively; the specific surface area, average pore volume and average pore size of IHA are 2.96 ± 0.08 m^2^·g^−1^, 0.0356 ± 0.0002 cm^3^·g^−1^ and 29.21 ± 0.04 nm, respectively; and the specific surface area, average pore volume and average pore size of IHA/TM are, respectively, are 9.90 ± 0.10 m^2^·g^−1^, 0.0438 ± 0.0002 cm^3^·g^−1^ and 17.67 ± 0.06 nm. The results show that the specific surface area of the IHA/TM composite particles were 1.23 times that of TM and 3.35 times that of IHA. It can be seen that the pore size of the synthetic material is between 2 and 50 nm, and the isotherms of the three substances belong to the adsorption curve of the IV-type mesoporous material and have an obvious hysteresis loop, indicating that the material has a good mesoporous structure. IHA/TM composite particles are beneficial for material dispersion, which produces more adsorption sites and adsorption interfaces in IHA/TM composite particles so that more Fe^2+^ and Mn^2+^ can be adsorbed, thus improving the performance of iron and manganese removal.

The SEM-EDS scanning electron microscope images of TM, IHA, and IHA/TM are shown in Figure 6b–d. The directional complexation of tourmaline particles forms a rough IHA/TM composite on the surface of IHA, and the small and dispersed tourmaline particles generate a large number of voids on the surface of the composite material, which increases the specific surface area, thereby improving the adsorption properties of IHA/TM composites. Among the main elements of the IHA/TM composite particles shown by EDS (as shown in Figure 6d), Al, Fe, B, O and Si are the main constituent elements of TM, while IHA has carboxyl and phenolic hydroxyl functional groups, which endow the composite material C, O, H and N elements (as shown in Figure 6e), and form metal complexes with metal ions such as Al and Fe in TM.

The functional groups and structures of TM, IHA and TM/IHA were analyzed by means of the unique infrared spectra of different compounds. As can be seen from Figure 6e, the absorption bands at 484 cm^−1^ are attributed to the bending vibration of Si-O, and the absorption bands at 710 cm^−1^ and 775 cm^−1^ are attributed to the bending stretching of M-O (M=Fe or Al). The peaks at 972 cm^−1^ and 1034 cm^−1^ correspond to O-Si-H stretching and bending vibration of O-Si-O, respectively. The absorption bands at 620 cm^−1^, 910 cm^−1^, 1080 cm^−1^, 1400 cm^−1^ and 1580 cm^−1^ are attributed to the bending vibration of C-O=C, the bending vibration of epoxy groups, C-O stretching, -OH stretching and C=O stretching vibration. It can be seen that IHA contains a large number of carbonyl and hydroxyl functional groups, which have the ability to adsorb heavy metals. It can be seen from the IHA/TM composite particle spectrum that the active functional groups of TM and IHA are retained in the preparation process of composite particles, so the adsorption of iron and manganese ions by the IHA/TM composite particles is not affected. In the IHA/TM composite particle spectrum, the vibration peaks of C=O and O-H become weaker, which may be due to the dehydration and decarboxylation of IHA during the calcination and the preparation of IHA/TM composite particles.

It can be seen from Figure 6f that, compared with the diffraction peak of the standard card of TM (PDF#85-1810), the diffraction peak of the tourmaline used in the experiment is slightly shifted, which is caused by the different components of tourmaline in different regions. Compared with the diffraction peaks of TM, the diffraction peaks of the IHA/TM composite powder are almost identical, indicating that the original crystal structure of the raw material was not destroyed during the production process, and the IHA/TM composite material was successfully prepared. Lei et al. obtained similar conclusions in the crystal structure of modifying hydroxyapatite nanoparticles with humic acid obtained from HA-modified HAP, and modifying hydroxyapatite nanoparticles with humic acid improved the adsorption efficiency of heavy metal ions [39]. It can also be seen from Figure 6f that the relative intensity of diffraction peaks of IHA/TM composites is stronger than that of TM, and it was found that the enhanced peak is limestone. The reason may be that TM raw materials are accompanied by a small amount of limestone, and the molar hardness of TM (7.5) is much higher than that of limestone (3). Before XRD measurement, TM grinding leads to finer limestone grinding, and limestone is not reflected in the measurement. In the process of preparing IHA/TM composite particles from TM and IHA, adding hydrochloric acid to adjust the pH value leads to the dissolution of limestone. When the pH value is adjusted by adding NaOH subsequently, limestone is regenerated by the action of CO_2_ in the air, which is reproduced in the diffraction peaks of the composite powder [40].

### 3.3. Analysis of Dynamic Adsorption Influence Factors

#### 3.3.1. Effect of the Adsorption Column Height on the Penetration Curve

**Figure 7:**
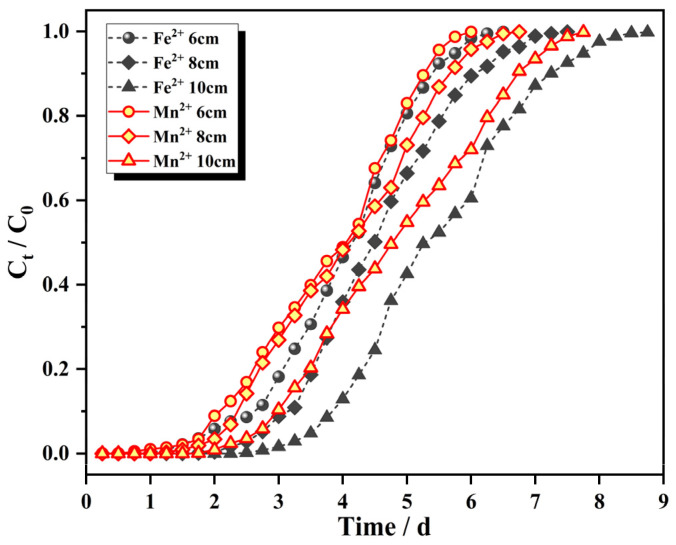
Breakthrough curves for different filler bed heights.

Figure 7 shows adsorption penetration curves for Fe^2+^ and Mn^2+^ with the initial influent concentrations of 25 mg/L and 10 mg/L, respectively, flow rates of 2.0 mL/min, and column bed depths of 6 cm (72 g), 8 cm (96 g) and 10 cm (120 g). The adsorption mass transfer parameters are shown in Table 2.

Figure 7 shows the characteristic “S” profile produced in an ideal adsorption system. With increasing operating time, the dynamic column effluent concentrations of Fe^2+^ and Mn^2+^ gradually increased until a dynamic equilibrium was reached. With increasing masses of IHA/TM composite particles, the slope of the curve gradually decreased, the breakthrough curve shifted to the right, the length of the mass transfer zone increased, and the equilibration time was prolonged. In other words, the breakthrough time and saturation time of the fixed bed were positively correlated with the bed height because the absorption capacity depended largely on the amount of absorbent available. When the bed height was higher, the increase in adsorbent mass provided a larger area for use. Then, more surface active sites were provided, the adsorption time was prolonged, the mass transfer zone was also enlarged, and the contact time between the solution and IHA/TM was prolonged, which enabled more effective adsorption of Fe^2+^ and Mn^2+^ ions [41]. When the amount of IHA/TM was small, the bed height was lower, the space resistance was smaller, the contact time between the solution and adsorbent was shorter, and complete adsorption by IHA/TM was not realized.

As seen from Table 2, the total amount adsorbed was positively correlated with the amount of adsorbent added, and the saturation adsorption amount was negatively correlated with the adsorbent dosage used; this was due to overcrowding between adsorbent particles when the amount of adsorbent was too large, inducing overlap covering the active sites, and the solution flowing through the adsorbent surface was unable to reach the maximum saturation capacity. The higher the bed height, the greater the loss, and the removal rate for Fe^2+^ and Mn^2+^ ions no longer increased, so the saturation adsorption capacity q_e_ decreased with increasing bed height. Therefore, neither low nor high bed heights were conducive to the dynamic adsorption of IHA/TM composite particles. If the bed height was too low, the penetration point was reached before the expected treatment effect was achieved, and if the bed height was too high, the removal of Fe^2+^ and Mn^2+^ was slow, and the effective utilization rate of the adsorbent was also reduced.

#### 3.3.2. Effect of Inlet Flow Rate on the Penetration Curve

**Figure 8:**
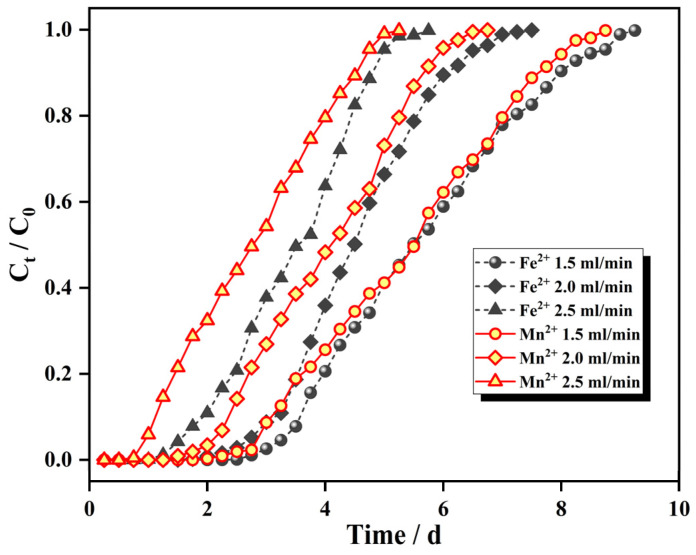
Breakthrough curves at different flow rates.

The Fe^2+^ ion influent concentration was 25 mg/L, the Mn^2+^ ion influent concentration was 10 mg/L, the column bed height was 8 cm, the influent water flow rate was changed, the adsorption breakthrough curve is shown in Figure 8, and the adsorption mass transfer parameters are shown in Table 2.

As seen from Figure 8, each ion penetration curve moves from right to left with increasing flow rate, the same ion penetration point and depletion point are advanced in turn, the adsorption equilibrium time is shortened in turn, and the slope of the curve increases accordingly; this arises because, with the increase in the flow rate, the transport resistance of the pollutants into the IHA/TM surface decreases, and the dwell time of the water sample in the adsorption bed column is shortened, shortening the contact times of ions in the water sample with the adsorbent, and adsorption is weakened, indicating that high adsorption velocities negatively affect the transfer effect of the IHA/TM.

As seen from Table 2, the dynamic adsorption capacity of the dynamic column for Fe^2+^ and Mn^2+^ ions was the highest at an inlet water flow rate of 2.0 mL/min, and both larger and smaller inlet water flow rates were unfavorable for dynamic adsorption by the IHA/TM composite particles. This is because the short residence times of the water sample at large inlet flow rates shortened the contact times between the IHA/TM composite particles and the water sample, which was unfavorable for the adsorption and diffusion of Fe^2+^ and Mn^2+^ ions and had a negative impact on the mass transfer speed of the IHA/TM. At a low flow rate, the dwell time of the IHA/TM in the bed column increased, and the adsorption efficiency was relatively high. However, the volume of water sample treated per unit time was small, longitudinal backmixing of the liquid phase easily occurred in the column, and the effective utilization of the IHA/TM was low [42]. Therefore, to ensure a certain adsorption capacity, the flow rate should be comprehensively determined by the column length, column diameter and other factors.

#### 3.3.3. Influence of Inlet Water Concentration on the Penetration Curve

**Figure 9:**
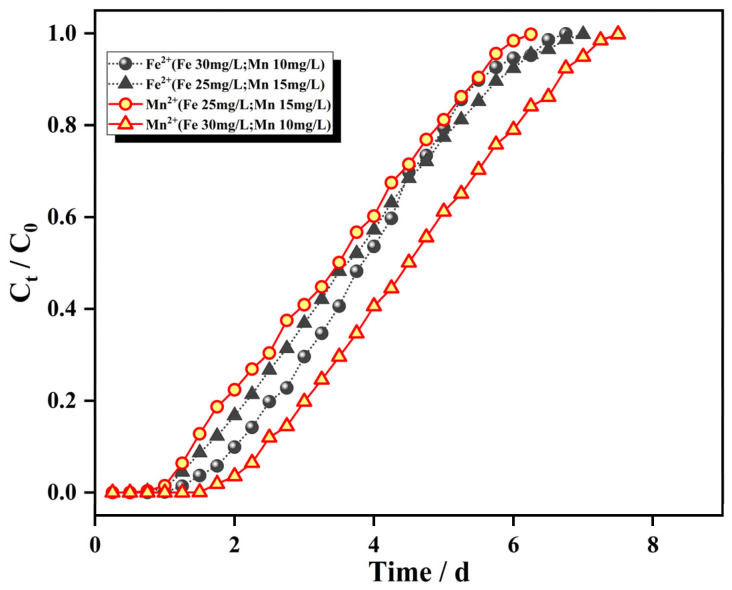
Breakthrough curves for different initial concentrations.

Figure 9 shows the adsorption breakthrough curve for a flow rate of 2.0 mL/min, bed column height of 8 cm, and that the influent concentration of only one ion, Fe^2+^ or Mn^2+^, was changed (Fe^2+^ from 25 mg/L to 30 mg/L; Mn^2+^ from 10 mg/L to 15 mg/L). The adsorption mass transfer parameters are shown in Table 2.

As seen from Figure 9, the patterns for the adsorption of Fe^2+^ and Mn^2+^ by the IHA/TM composite particles were similar after increasing the initial mass concentrations of Fe^2+^ and Mn^2+^. The slope of the penetration curve for each ion increased significantly, the penetration curves gradually shifted to the left, and the time to reach adsorption equilibrium was advanced, which indicates that the higher inlet water concentration provided a greater driving force for the adsorption of Fe^2+^ and Mn^2+^ by the IHA/TM composite particles. The activation energy of the adsorption reaction was enhanced. The activation energy for adsorption allowed the faster occupation of adsorption sites on the surface and resulted in a larger surface coverage; this made adsorption faster, more pollutants came into contact with the adsorbent per unit of time, adsorption saturation was achieved more readily and the penetration time and saturation time were greatly shortened with increased influent concentration due to the limited total number of ion adsorption sites [43]. Table 2 clearly shows that the dynamic adsorption capacity of the IHA/TM composite particles for both Fe^2+^ and Mn^2+^ ions increased with increasing influent concentration, which arose because the higher influent ion concentration increased the driving force and overcame the mass transfer potential [44].

### 3.4. Adsorption Kinetic Analysis

The adsorption penetration curves for Fe^2+^ and Mn^2+^ adsorption by the IHA/TM composite particles were fitted under different adsorbent filling heights, different inlet flow rates and different initial concentrations by applying the Adams–Bohart model, Thomas model and Yoon–Nelson model, respectively, and the results are shown in Table 3 and Table 4.

Table 3 and Table 4 show that, for the Adams–Bohart model, the mass transfer rate constant *K*_AB_ and the ion adsorption saturation concentration *N*_0_ decreased with increasing the bed depth for both Fe^2+^ and Mn^2+^; *K*_AB_ decreased, but *N*_0_ increased with increasing initial concentrations of Fe^2+^ and Mn^2+^ ions; with increasing flow rate, the mass transfer rate constant *K*_AB_ for both Fe^2+^ and Mn^2+^ ions gradually increased, while N_0_ first increased, then decreased, and then reached a maximum at a flow rate of 2.0 mL/min. The Adams–Bohart model is commonly used to describe the initial stage of the penetration curve, but it ignores the mass transfer and membrane diffusion resistance inside the particles and assumes that adsorption equilibrium is reached instantaneously [45]. The correlation coefficients *R*^2^ resulting after fitting the adsorption data for Fe^2+^ and Mn^2+^ were 0.645~0.837 and 0.623~0.862, respectively, and were not suitable for describing the dynamic adsorption process of IHA/TM composite particles; however, based on the variations in their correlation parameters, it can be concluded that the kinetics for the initial stage of adsorption in the fixed column system were dominated by external mass transfer [46].

For the Yoon–Nelson model, the rate constant K_YN_ and the theoretical unit adsorption volume q_YN_ decreased with increasing the bed depth for both Fe^2+^ and Mn^2+^, but the half penetration rate *τ* (*τ* is the time required for 50% adsorbate breakthrough (min)) of the adsorbent increased; the increase in the bed height extended the penetration time of Fe^2+^ and Mn^2+^ ions, while the increase in the adsorbent mass increased the resistance between Fe^2+^ and Mn^2+^ ions and the adsorbent, so *K*_YN_ decreased and τ increased. With increases in the initial concentrations of Fe^2+^ and Mn^2+^ ions, the rate constant *K*_YN_ and the half penetration rate *τ* of the adsorbent decreased, but the theoretical unit adsorption amount q_YN_ increased; with the increased flow rate, the rate constant *K*_YN_ increased, and the half penetration rate τ of the adsorbent decreased. The theoretical unit adsorption amount q_YN_ first increased and then decreased and reached a maximum at a flow rate of 2.0 mL/min.

For the Thomas model, the mass transfer rate *K*_Th_ decreased with increasing bed height and concentration and increased with increasing flow rate for both Fe^2+^ and Mn^2+^, while the maximum adsorption capacity *q*_0_ of the IHA/TM decreased with increasing bed depth, increased with increasing concentration and was also at a maximum with a flow rate of 2.0 mL/min. It was shown that changing the bed height, inlet flow rate and concentration gradient of each ion affected the adsorption processes in dynamic columns. This result arose because an increased bed height led to more adsorption sites and extended the total operating time of the fixed bed [47]. The concentrations of Fe^2+^ and Mn^2+^ ions increased, and the concentration difference between IHA/TM and Fe^2+^ and Mn^2+^ increased, which increased the mass transfer driving force. Fe^2+^ and Mn^2+^ were more easily adsorbed by IHA/TM, and thus, *q_0_* was increased. An excessively large flow rate shortened the contact time between the pollutants and adsorbent, the utilization rate for active sites in the adsorbent decreased, and *q*_0_ decreased.

For the dynamic adsorption of Fe^2+^ and Mn^2+^ by the IHA/TM composite particles, the correlation coefficient *R^2^* (≥0.964) obtained by the Thomas model fitting is generally closer to 1 than that obtained by the Adams–Bohart model (≥0.623) and the Yoon–Nelson model (≥0.901), indicating that the Thomas model can describe the kinetic characteristics of the dynamic adsorption of Fe^2+^ and Mn^2+^ ions by the IHA/TM composite particles.

### 3.5. Desorption and Reusability of Insoluble Humic Acid/Tourmaline Composite Particles

Table 5 shows that, with increasing regeneration time, the adsorption capacity of the IHA/TM composite adsorbent for Fe^2+^ and Mn^2+^ decreased. This may be due to insufficient elution, making some Fe^2+^ and Mn^2+^ occupy the adsorption sites, or the complexation reaction between Fe^2+^ and Mn^2+^ with the IHA/TM composite adsorbent making Fe^2+^ and Mn^2+^ not fully desorbed. However, after five regenerations, the regeneration rate of Fe^2+^ and Mn^2+^ by the IHA/TM composite adsorbent decreased by only 10.1% and 12.7%, indicating that the IHA/TM composite adsorbent has a strong regeneration ability.

In order to further determine the residual amount of iron and manganese on the materials, the EDS spectrum scanning of the TM/IHA composite particles was carried out between the regeneration cycles, and the relative content of iron and manganese on the material was determined. Table 6 shows that the relative content of iron and manganese increases with the increase in regeneration times. It further explained that the elution solution could not completely desorb Fe^2+^ and Mn^2+^ on TM/IHA composite particles, resulting in the decrease in the adsorption capacity of the IHA/TM composite adsorbent for Fe^2+^ and Mn^2+^.

## 4. Conclusions

(1) The optimal preparation conditions of the IHA/TM composite adsorbent were as follows: the mixing ratio of IHA and TM was 2:3, the mixing time was 18 h, the calcination temperature was 330 °C and the calcination time was 90 min. The prepared IHA/TM composite adsorbent not only has the multifunctional groups properties of organic insoluble humic acid, but also has the thermoelectric and spontaneous polarization effects of inorganic silicate tourmaline, and has a good adsorption efficiency for high-concentration iron- and manganese-contaminated mine wastewater.
(2) The filling height of the IHA/TM in the dynamic column, the inlet water flow rate and the initial concentrations of Fe^2+^ and Mn^2+^ all affected the penetration process of the dynamic adsorption of the IHA/TM composite particles. With an increase in the column bed height, the decrease in the flow rate, and the decrease in the initial concentration of Fe^2+^ and Mn^2+^, the penetration curves of each ion moved from left to right and the adsorption equilibration time lengthened in turn. At a filling height of 8 cm and an inlet water flow rate of 2 mL/min, the maximum dynamic adsorption capacities of the dynamic column for Fe^2+^ and Mn^2+^ with the initial concentrations of 25 mg/L and 10 mg/L, respectively, were 2.348 mg/g and 1.025 mg/g.
(3) For the dynamic adsorption of Fe^2+^ and Mn^2+^ by the IHA/TM composite adsorbent, the correlation coefficient *R*^2^ (>0.96) of the Thomas model after the adsorption of Fe^2+^ and Mn^2+^ and the theoretical unit adsorption capacity obtained from the analysis were closer to the actual value, so the Thomas model is more suitable for describing the dynamic adsorption process of the IHA/TM composite particles.

## Figures and Tables

**Table 1:** Comparison of the adsorption effect of different adsorption materials on iron and manganese ions.

Adsorbent Material	pH	*q*_e_ of Fe^2+^ (mg/g)	*q*_e_ of Mn^2+^ (mg/g)	References
Modified silica alumina sand	6.5		0.319	[35]
Rice husk ash	5.0	4.19		[36]
Modified fly ash	3.0	4.8	0.81	[37]
Corn cob	5.5	2.5	2.3	[14]
Y-type zeolite	6.5	0.023	0.015	[15]
Yeast	5–6	4.46	2.23	[38]
IHA/TM composite particles	6.0	5.318	3.106	This research

**Table 2:** Adsorption parameters of IHA/TM at different influencing factors.

Ions	*H* (cm)	*Q* (mL/min)	*C* (mg/L)	*T*_b_ (d)	*T*_e_ (d)	*q*_total_ (mg)	*q*_e_ (mg/g)
Fe^2+^	6	2	25	2.0	5.75	196.24	2.726
	8	2	25	2.75	6.5	225.38	2.348
	10	2	25	3.5	7.75	264.49	2.204
	8	1.5	25	3.5	7.75	222.77	2.321
	8	2.5	25	2.25	6.25	214.65	2.236
	8	2	30	1.75	6.25	257.04	2.678
Mn^2+^	6	2	10	2.0	5.5	75.72	1.052
	8	2	10	2.25	6.0	95.66	1.025
	10	2	10	2.75	7.25	100.85	0.840
	8	1.5	10	3	7.5	83.12	0.866
	8	2.5	10	1.5	5.75	81.65	0.851
	8	2	15	1.25	5.75	118.74	1.237

**Table 3:** Model parameters for the adsorption of Fe^2+^ on IHA/TM.

Test Parameters			Adams–Bohart Model			Thomas Model			Yoon–Nelson Model			
*C*_0_/ (mg/L)	*Q*/ (mL/min)	*H*/ cm	*K*_AB_/ ×10^−5^	*N*_0_/ ×10^4^ mg/L	*R* ^2^	*K*_Th_	*q*_0_/ (mg/g)	R^2^	*K*_YN_× 10^−2^/ min^−1^	*τ*/ min	*q*_YN_/ (mg/g)	*R* ^2^
25	2	6	2.582	6.529	0.837	0.052	2.477	0.983	0.129	5572	3.869	0.955
25	2	8	2.432	5.607	0.7744	0.050	2.184	0.989	0.126	6512	3.392	0.970
25	2	10	2.158	5.292	0.749	0.044	2.126	0.987	0.111	7975	3.323	0.967
25	1.5	8	1.773	5.231	0.645	0.035	2.077	0.964	0.088	8311	3.247	0.903
25	2.5	8	2.815	5.360	0.707	0.057	2.029	0.983	0.142	4870	3.171	0.932
30	2	8	1.781	5.960	0.690	0.037	2.200	0.976	0.111	5501	3.458	0.917

**Table 4:** Model parameters for the adsorption of Mn^2+^ on IHA/TM.

Test Parameters			Adams–Bohart Model			Thomas Model			Yoon–Nelson Model			
*C*_0_/ (mg/L)	*Q*/ (mL/min)	*H*/ cm	*K*_AB_/ ×10^−5^	*N*_0_/ ×10^4^ mg/L	*R* ^2^	*K*_Th_	*q*_0_/ (mg/g)	R^2^	*K*_YN_× 10^−2^/ min^−1^	*τ*/ min	*q*_YN_/ (mg/g)	*R* ^2^
10	2	6	6.586	2.436	0.862	0.123	0.876	0.987	0.123	5258	1.461	0.928
10	2	8	5.919	2.258	0.665	0.112	0.871	0.992	0.119	5740	1.196	0.937
10	2	10	5.554	1.871	0.718	0.105	0.746	0.984	0.105	6995	1.166	0.924
10	1.5	8	4.463	1.987	0.728	0.087	0.759	0.987	0.087	7795	1.186	0.930
10	2.5	8	6.045	1.958	0.623	0.127	0.660	0.971	0.126	3958	1.031	0.901
15	2	8	3.155	2.791	0.681	0.066	0.986	0.982	0.099	4928	1.540	0.912

**Table 5:** Regeneration capacity of the IHA/TM composite adsorbent.

Cycles	Fe^2+^		Mn^2+^	
	Adsorption Capacity (mg/g)	Regeneration Rate (%)	Adsorption Capacity (mg/g)	Regeneration Rate (%)
1	2.310	98.4	1.002	97.8
2	2.270	96.7	0.980	95.6
3	2.188	93.2	0.951	92.8
4	2.146	91.4	0.916	89.4
5	2.073	88.3	0.872	85.1

**Table 6:** Relative contents of various elements in TM/IHA composite particles after desorption with nitric acid and scanning by EDS.

Cycles	C	O	N	Al	Si	B	Fe	Mg	S	Mn
1	46.13	30.95	0.74	2.28	1.54	15.57	0.94	1.43	0.37	0.05
2	46.88	30.48	0.77	2.37	1.49	15.26	1.02	1.27	0.36	0.10
3	46.29	31.64	0.79	2.18	1.38	14.97	1.17	1.02	0.38	0.18
4	47.32	30.65	0.80	2.26	1.11	15.08	1.24	0.91	0.35	0.28
5	46.91	30.86	0.76	2.08	0.97	15.47	1.38	0.86	0.36	0.35

## Data Availability

Data can be obtained from corresponding authors upon reasonable request.

## References

[1] Li X., Yu X., Liu L., Yang J., Liu S., Zhang T. (2021). Preparation, characterization serpentine-loaded hydroxyapatite and its simultaneous removal performance for fluoride, iron and manganese. RSC Adv..

[2] Farrag A.E.H.A., Abdel M.T., Mohamed A.M.G., Saleem S.S., Fathy M. (2017). Abu zenima synthetic zeolite for removing iron and manganese from assiut governorate groundwater, egypt. Appl. Water. Sci..

[3] Akbar N.A., Aziz H.A., Adlan M.N. (2015). Potential of high quality limestone as adsorbent for iron and manganese removal in groundwater. Appl. Mech. Mater..

[4] Jusoh A.B., Cheng W.H., Low W.M., Nora’Aini A., Noor M. (2005). Study on the removal of iron and manganese in groundwater by granular activated carbon. Desalination.

[5] Zhang L., Zhou T. (2015). Drought over East Asia: A Review. J. Clim..

[6] Sung B., Chu K., Yun S. (2015). Removal of iron and manganese ions from abandoned neutral or alkaline mine drainage via ozone oxidation and micro-sand filtration: A pilot-scale operation. Desalin. Water Treat..

[7] Melo C., Riella H., Kuhnen N. (2014). Adsorption of iron and manganese from acid mine drainage using 4A-zeolite synthesised from waste with high kaolin concentrations. Int. J. Environ. Pollut..

[8] Bai Z. (2014). Engineering application of the removal of iron and manganese from mine water. Ind. Water Treat..

[9] Ren H., Zhu S., Wang X., Liu Y., Cao L. (2020). Study on Issues and Countermeasures in Coal Measures Mine Water Resources Exploitation and Utilization. China Coal Geol..

[10] Ellis D., Bouchard C., Lantagne G. (2000). Removal of iron and manganese from groundwater by oxidation and microfiltration. Desalination.

[11] Li X., Wang Q., Liu L., Liu S. (2021). The performance of calcined serpentine to simultaneously remove fluoride, iron and manganese. Water Supply.

[12] Adekola F.A., Hodonou D.S.S., Adegoke H.I. (2016). Thermodynamic and kinetic studies of biosorption of iron and manganese from aqueous medium using rice husk ash. Appl. Water Sci..

[13] Su X., Hu J., Zhang J. (2020). Investigating the adsorption behavior and mechanisms of insoluble Humic acid/starch composite microspheres for metal ions from water. Colloids Surf. A.

[14] Nassar M. (2006). Adsorption of Fe^3+^ and Mn^2+^ from GroundWater onto Maize Cobs Using BatchAdsorber and Fixed Bed Column. Sep. Sci. Technol..

[15] Kwakye-Awuah B., Sefa-Ntiri B., Von-Kiti E., Nkrumah I., Williams C. (2019). Adsorptive Removal of Iron and Manganese from Groundwater Samples in Ghana by Zeolite Y Synthesized from Bauxite and Kaolin. Water.

[16] Fierascu R.C. (2021). Recent Progress in the Application of Hydroxyapatite for the Adsorption of Heavy Metals from Water Matrices. Materials.

[17] Panek R., Medykowska M., Winiewska M. (2021). Simultaneous Removal of Pb^2+^ and Zn^2+^ Heavy Metals Using Fly Ash Na-X Zeolite and its Carbon Na-X(C) Composite. Materials.

[18] Nakamura T., Kubo T. (2011). Tourmaline group crystals reaction with water. Ferroelectrics..

[19] Liao G., Zhao W., Li Q., Pang Q., Xu Z. (2017). Novel Poly(acrylic acid)-modified Tourmaline/Silver Composites for Adsorption Removal of Cu(II) ions and Catalytic Reduction of Methylene Blue in Water. Chem. Lett..

[20] Wei Z., Jin C., Wang Y., Lin H., Sythari P., Wei D. (2020). Preparation of tourmaline bamboo-charcoal ceramic composites and its adsorption properties for Cr(VI). New Chem. Mater..

[21] Chianese S., Fenti A., Iovino P., Musmarra D., Salvestrini S. (2020). Sorption of organic pollutants by humic acids: A review. Molecules.

[22] Leone V., Iovino P., Capasso S., Trifuoggi M., Musmarra D. (2018). Sorption of benzene derivatives onto insolubilized humic acids. Chem. Pap..

[23] Hu D., Zhang F., Zhou Y., Zhang H., Ke X. (2019). Effect of humic acid load hydroxyapatite on the adsorption of Cd^2+^ in wastewater. Acta Sci. Circumstantiae.

[24] Zhang X., Zhang P., Wu Z., Zhang L., Zeng G., Zhou C. (2013). Adsorption of methylene blue onto humic acid-coated Fe_3_O_4_ nanoparticles. Colloids Surf. A..

[25] Liu L., Zhang T., Yu X., Mkandawire V., Ma J., Li X. (2022). Removal of Fe^2+^ and Mn^2+^ from Polluted Groundwater by Insoluble Humic Acid/Tourmaline Composite Particles. Materials.

[26] Dong Y., Lin H. (2017). Competitive adsorption of Pb(II) and Zn(II) from aqueous solution by modified beer lees in a fixed bed column. Process Saf. Environ. Prot..

[27] Emmanuel D., Siewe J., Njopwouo D. (2015). A fixed-bed column for phosphate removal from aqueous solutions using an andosol-bagasse mixture. J. Environ. Manag..

[28] Rangabhashiyam S., Suganya E., Selvaraju N. (2016). Packed bed column investigation on hexavalent chromium adsorption using activated carbon prepared from swietenia mahogani fruit shells. Desalin. Water Treat..

[29] Keshtkar A., Kafshgari F., Mousavian M. (2012). Binary biosorption of uranium(vi) and nickel(ii) from aqueous solution by ca-pretreated cystoseira indica in a fixed-bed column. J. Radioanal. Nucl. Chem..

[30] Chen Y., Wang S., Li Y. (2020). Adsorption of Pb(II) by tourmaline-montmorillonite composite in aqueous phase. J. Colloid Interface Sci..

[31] Wang F., Zhang X., Liang J., Fang B., Zhang H., Zhang Y. (2018). Phase transformation and microstructural evolution of black tourmaline mineral powders during heating and cooling processes. Ceram. Int..

[32] Wang F., Meng J., Liang J., Fang B., Zhang H. (2019). Insight into the thermal behavior of tourmaline mineral. JOM.

[33] Seki H., Suzuki A. (1995). Adsorption of heavy metal ions onto insolubilized humic acid. J. Colloid Interface Sci..

[34] Zhao W., Ren B., Hursthouse A., Jiang F. (2020). The adsorption of Mn(II) by insolubilized humic acid. Water Sci. Technol..

[35] Bai L., Zhao X., Ding J., Li G., Liang H. (2020). Study on removal of manganese from modified silica alumina sand. Water Wastes Eng..

[36] Zhang Y., Lu Y., Zhao J., Jiang Z., Cao B., Song Q., Su G., Li M. (2015). Dynamic adsorption of iron ions in groundwater by rice husk ash. J. Northeast. Agric. Univ..

[37] Wang J., Cui Y., Bai F., Chen M. (2013). Removal of Fe(III) and Mn(II) from Aqueous Solutions Using Modified Fly Ash. J. Agric. Environ. Sci..

[38] Zheng R. (2013). Adsorption Characteristic Study of Fe(II) and Mn(II) from Groundwater Using Biosorbents. Master’s Thesis.

[39] Yang L., Wei Z., Zhong W., Cui J., Wei W. (2016). Modifying hydroxyapatite nanoparticles with humic acid for highly efficient removal of Cu(II) from aqueous solution. Colloids Surf. A Physicochem. Eng. Aspects..

[40] Xue G., Luo X., Srinivasakannan C., Zheng L., Miao Y., Duan X. (2019). Effective removal of organic dye and heavy metal from wastewater by tourmaline/graphene oxide composite nano material. Mater. Res. Express.

[41] Vilvanathan S., Shanthakumar S. (2017). Column adsorption studies on nickel and cobalt removal from aqueous solution using native and biochar form of Tectona grandis. Environ. Prog. Sustain. Energy..

[42] Du Z., Jia M., Men J. (2014). Dynamic performance of Cs^+^ adsorption from aqueous solution with polyacrylonitrile-potassium titanium hexacyanoferrate(II) spherical composite adsorbent. J. Cent. South Univ. (Sci. Technol.).

[43] Kizito S., Wu S., Wandera S.M., Guo L., Dong R. (2016). Evaluation of ammonium adsorption in biochar-fixed beds for treatment of anaerobically digested swine slurry: Experimental optimization and modeling. Sci. Total Environ..

[44] Agrawal P., Bajpai A.K. (2011). Dynamic Column Adsorption Studies of Toxic Cr(VI) Ions onto Iron Oxide Loaded Gelatin Nanoparticles. J. Dispers. Sci. Technol..

[45] Mahdi Z., Yu Q.J., El Hanandeh A. (2018). Removal of lead(II) from aqueous solution using date seed-derived biochar: Batch and column studies. Appl. Water Sci..

[46] Aksu Z., Gönen F. (2004). Biosorption of phenol by immobilized activated sludge in a continuous packed bed: Prediction of breakthrough curves. Process Biochem..

[47] Jellali S., Diamantopoulos E., Haddad K., Anane M., Durner W., Mlayah A. (2016). Lead removal from aqueous solutions by raw sawdust and magnesium pretreated biochar: Experimental investigations and numerical modelling. J. Environ. Manag..

